# Ischemic stroke in young Asians caused by spontaneous cervical artery dissection may be due to slightly increased homocysteine

**DOI:** 10.3389/fneur.2025.1527896

**Published:** 2025-03-10

**Authors:** Zijun Lin, Shuhan Huang, Wei Li

**Affiliations:** ^1^Department of Neurology, Army Medical Center of PLA, Army Medical University, Chongqing, China; ^2^Department of Neurology, The First Affiliated Hospital of Chongqing Medical University, Chongqing, China

**Keywords:** spontaneous cervical artery dissection, homocysteine, ischemic stroke, risk factors, Asian young adults

## Abstract

**Background:**

Spontaneous cervical artery dissection (sCAD) is a non-atherosclerotic vascular disease among young and middle-aged individuals of unknown etiology that is recognized as a cause of ischemic stroke. Total plasma homocysteine (tHcy) is associated with an increased risk of sCAD, but the precise mechanism and level of tHcy remain unclear.

**Methods:**

Fasting tHcy levels were determined in 296 patients with a first ischemic stroke due to sCAD (*n* = 159) and in age-/gender-matched hospital-based controls (*n* = 137) within 24 h after the onset of symptoms.

**Results:**

The mean age of sCAD patients with ischemic stroke and controls was 45.6 years; 61.0% of the cases and controls were male. The prevalence rates of hypertension, diabetes mellitus, and hyperlipidemia in sCAD patients were significantly increased. Fasting tHcy levels in sCAD patients were significantly higher (12.81 ± 5.24 μmol/L, 95% CI: 11.79–13.89) than those in controls (10.21 ± 3.33 μmol/L, 95% CI: 9.92–11.89, *p* < 0.001). Compared with the lowest homocysteine quartile, the quartile between 12.1 and 14.54 μmol/L was significantly associated with sCAD, with an adjusted odds ratio of 4.7. The adjusted odds ratio was 5.02 (95% CI: 1.91–13.39, *p* = 0.001) for every 1 μmol/L increase in log homocysteine according to the logistic regression model. Although sCAD occurred more often in winter than in other seasons (*p* = 0.02), tHcy levels in sCAD were not significantly different in terms of seasonal variation (*p* > 0.05).

**Conclusion:**

Our results suggest that mildly increased tHcy may be a predisposing risk factor for ischemic stroke in young Asians caused by sCAD and that the relationship between them is significant; however, the precise mechanism requires further study. This result also supports the screening of fasting tHcy in young Asian adults for early intervention and control of tHcy levels, which plays an important role in early clinical prediction and intervention of sCAD.

## Introduction

In young to middle-aged adults, cervical artery dissection (CAD) is recognized as a major cause of ischemic stroke, accounting for approximately 25% of ischemic strokes in individuals under 55 years of age. CAD can be divided into traumatic carotid artery dissection and spontaneous cervical artery dissection (sCAD) according to the etiology (1). Despite improvements in clinical awareness and the development of non-invasive investigational tools, the pathogenesis of CAD is still not well understood. At present, identified CAD-related risk factors include connective tissue disorders (2), migraine (3), recent infection (4), and trauma (5). However, some sCAD cases are spontaneous without any clear risk factors (1). Pathophysiology suggests that underlying arterial susceptibility and primary disease of the arterial wall are possible triggers for sCAD (6). Mild hyperhomocysteinemia can promote atherosclerosis in the cerebral arteries, which is a risk factor for ischemic stroke (7). Case-control studies have reported that sCAD is significantly associated with total plasma homocysteine (tHcy); however, patients with sCAD typically have no or minimal atherosclerosis (8). These findings suggest that mild-hyper tHcy may predispose patients to sCAD. However, the association between tHcy level and sCAD is still an enigma. sCAD patients are younger than other stroke patients, so the effects of long-term exposure to these mild tHcy levels have not been studied.

Only two studies have reported that sCAD is associated with mild hyperhomocysteinemia (hyper-Hcy) (9, 10). However, the exact level of tHcy has not been determined. Therefore, we conducted a case-control study to evaluate which levels of tHcy may be associated with sCAD.

## Methods

### Data collection of cases and controls

Patients with the first-ever ischemic stroke were taken from the Army Medical Center of the People’s Liberation Army of China, and consecutive patients were recruited between February 2010 and November 2023.

The patients were (1) between 18 and 55 years old. (2) First ischemic stroke caused by sCAD. (3) The levels of tHcy were measured within 24 h after the onset of stroke symptoms to avoid the influence of the progressive increase in tHcy levels in the acute phase of stroke (11). (4) All patients underwent a comprehensive assessment, which included medical history assessment, neurological examination, routine blood examinations at admission, sCAD-related risk factor assessment, computed tomography angiography (CTA), magnetic resonance angiography (MRA), or digital subtraction angiography (DSA) (12). (5) Detection of sCAD based on vascular territory maps. The double-lumen sign (false lumen or intimal flap), luminal narrowing with the “string sign,” and gradual tapering, which eventually leads to the total occlusion of the lumen (flame sign), are considered reliable angiographic manifestations of sCAD.

The control group included age- and sex-matched Asian patients admitted to the same hospital for acute conditions other than ischemic stroke.

### Exclusion criteria

(1) Patients with a history of known ischemic heart disease, ischemic stroke, or peripheral vascular disease were excluded. (2) Cervical artery dissections were classified as sCAD when they occurred spontaneously. Patients with clear head or neck trauma or external force were excluded from this study. (3) Pregnant patients and patients taking drugs that might affect homocysteine, folate metabolism, or vitamin B12 were also excluded.

### Determination of fasting tHcy levels

Fasting phlebotomy was performed, and blood samples were placed on ice and centrifuged within 6 h. After preprocessing the samples, fluorescence polarization immunoassay (FPIA) was used to measure the fasting levels of tHcy within 24 h after the onset of symptoms. Hyperhomocysteinemia was defined as fasting tHcy levels above 12.0 μmol/L.

### Risk factor assessment

Baseline demographic data were obtained for all included populations (cases and controls), and a medical history of conventional vascular risk factors (hypertension, diabetes mellitus, hyperlipidemia, and smoking) was collected. Pay attention to distinguishing the following stroke risk factors: current smoking status defined as smoking in the last 5 years; former smoking or quitting smoking defined as abstention from smoking for more than 5 years (13); hyperlipidemia was defined as patients with a past history of hyperlipidemia or fasting serum total cholesterol were more than 5.2 mmol/L. Diagnosis of diabetes mellitus is defined as having a past history of diabetes mellitus or fulfilling World Health Organization criteria for diabetes (14).

### Statistics analysis

Baseline differences between the two groups (patients with sCAD, stroke-free controls) in proportions were examined with the chi-square test for categorical data. For continuous data, the *t*-test was used to compare the differences in the mean values. The normality of data distribution was checked with skewness and kurtosis tests. Because homocyst(e)ine was positively skewed, a natural logarithmic transformation was used, and results were expressed as geometric means. A multinomial logistic regression model was used to test the association between age, sex, diabetes, hypertension, smoking, and hypercholesterolemia between the two groups. Then, we used logistic regression models to evaluate the associations between tHcy levels and patients with sCAD. Multivariate logistic regression models were constructed by adjusting for confounding factors with sCAD as the major outcome variable and tHcy as the main covariate. In the logistic regression model, tHcy and vascular-related risk factors are interaction terms (tHcy and diabetes mellitus, tHcy and hypertension, tHcy and hyperlipidemia) that are evaluated for interaction. We used chi-square analysis to test the distribution of sCAD across the four seasons. Results were considered significant if *p* < 0.05 and expressed as OR and its 95% confidence interval (CI). Statistical analysis was carried out with Prism 8 (College Station, Texas, United States).

## Results

A total of 317 consecutive patients participated in this study, and 21 (12%) were excluded from this analysis because cervical artery dissection was caused by a clear trauma. Therefore, the study group comprised 159 sCAD patients and 137 controls.

### Baseline characteristics between cases and controls

The clinical characteristics and demographics of cases and controls according to disease status and disease-related risk factors are summarized in Table 1. In this study, the control group was matched with the age and gender of the case group, so there were no statistically significant differences in age or gender composition. The mean age (*p* = 0.522) was 45.6 ± 7.19 years in the sCAD cases and 45.1 ± 9.26 years in the controls. The percentage of males (*p* = 1.0) was 61.0% in both groups.

**Table 1:** Baseline demographics, conventional vascular risk factors, and fasting plasma homocysteine levels in sCAD and controls.

	sCAD (*n* = 159)	Controls (*n* = 137)	Crude models	
			Ratio and 95% CI	*p* ^a^
Male, *n* (%)	97 (61.0%)	84 (61.0%)	…	1.0
Mean age, y (SD)	45.6 ± 7.19	45.1 ± 9.26	…	0.522
Smoking status, *n* (%)	62 (42.1%)	60 (43.8%)	0.89 (0.52 to 1.73)	0.673
Hypertension, *n* (%)	92 (57.9%)	31 (22.6%)	4.88 (2.62 to 9.41)	<0.001
Diabetes mellitus, *n* (%)	59 (32.1%)	16 (13.9%)	4.11 (1.76 to 9.32)	0.002
Hyperlipidemia, *n* (%)	133 (83.6%)	58 (42.3%)	5.67 (2.96 to 9.91)	<0.001
Mean total cholesterol, mmol/L	5.07 ± 1.27	4.16 ± 1.12	0.73 (0.22–0.92)	0.029
Mean triglyceride, mmol/L	1.53 ± 0.63	0.89 ± 1.10	0.69 (0.62–1.00)	0.004
Homocysteine, μmol/L				
Mean^b^	12.81	10.21		
Range	(5.0–60.3)	(1.4–24.9)		
95% CI	(11.79 to 13.89)	(9.92 to 11.89)	…	<0.001
Treat with antiplatelet drugs, *n*	116	…		
Treat with anticoagulant drugs, *n*	43	…		

sCAD, spontaneous cervical artery dissection; SD standard deviation. Regression models were adjusted for covariates.

a Chi-square test for categorical variables, unpaired *t*-test for continuous variables. The values in parentheses are percentages or 95% CI.

b Homocyst(e)ine levels expressed as geometric means.

The prevalence of conventional vascular risk factors, such as hypertension (57.9% vs. 22.6%, *p* < 0.001), diabetes mellitus (32.1% vs. 13.7%, *p* = 0.002), and hyperlipidemia (83.6% vs. 42.3%, *p* < 0.001), were significantly higher in sCAD cases than in controls. Among hyperlipidemia, the mean total cholesterol was significantly higher (5.07 ± 1.27 mmol/L, 95% CI: 0.22–0.92, *p* = 0.029), but no significant difference in mean triglyceride (5.07 ± 1.27 mmol/L, 95% CI: 0.62–1.00, *p* = 0.426). There was no significant difference in smoking status (42.1.6% vs. 43.8%, *p* = 0.673) between cases and controls. Mean levels of tHcy were significantly higher in patients with sCAD (12.81 ± 5.24 μmol/L, 95% CI: 11.79 to 13.89) compared with controls (10.21 ± 3.33 μmol/L, 95% CI: 9.92 to 11.89), and the difference was significant (*p* < 0.001).

### Risk of sCAD

**Figure 1:**
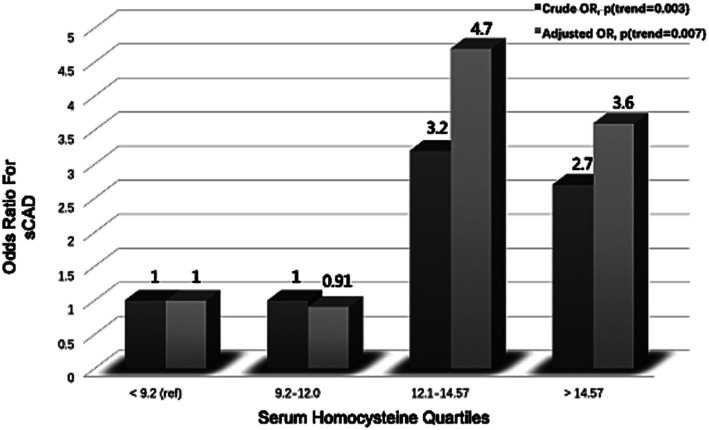
Association between serum homocysteine quartiles and risk of spontaneous cervical artery dissection (sCAD).

tHcy levels showed an independent relationship to the risk of sCAD (Figure 1). There was a significant difference in mean tHcy levels between sCAD and controls before and after adjustment for age and gender (*p* < 0.001). The crude odds ratio (OR) was 3.2 (95% CI: 1.3 to 8.7) when the quartile of homocysteine was between 12.1 and 14.57 μmol/L compared with the lowest quartile. After adjustment for age, sex, and vascular risk factors, the adjusted OR was 4.7 (95% CI: 1.9 to 13.3).

Logistic regression model analysis was performed with sCAD as the dependent variable and with continuous variables (homocysteine) and categorical variables (diabetes mellitus, hypertension, and hyperlipidemia) as independent variables. The above statistical results are summarized in Table 2. tHcy was considered to be an important independent risk factor, and the adjusted OR was 5.02 (95% CI: 1.91 to 13.39) for every 1 μmol/L increase in log homocysteine. The statistical results showed that hypertension was the most important independent risk factor of conventional vascular risk factors, with an adjusted OR of 3.89 (95% CI: 1.74 to 11.83).

**Table 2:** Associations of risk factors with sCAD based on logistic regression modeled.

Risk factor	Crude odds ratio	95% CI	Adjusted odds ratio	95% CI	*p*
Hypertension					
No	1.00		1.00		
Yes	4.97	2.64–9.38	3.89	1.74–11.83	0.007
Diabetes mellitus					
No	1.00		1.00		
Yes	3.99	1.72–9.18	1.61	1.52–8.28	0.029
Hyperlipidemia					
No	1.00		1.00		
Yes	4.28	2.79–9.14	2.56	2.51–11.69	0.041
Homocyst(e)ine	4.36	1.89–9.94	5.02^a^	1.91–13.39	0.001

a For every μmol/L increase in log homocyst(e)ine.

### Seasonal variation

**Figure 2:**
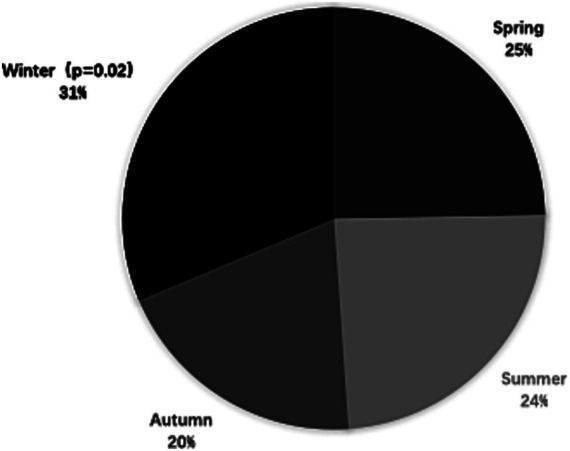
Frequencies of documented cases of spontaneous cervical artery dissection (sCAD) by season in China.

As shown in Figure 2, sCAD occurred more frequently in winter (31.3%; 95% CI: 27.2 to 36.6; *p* = 0.02) than spring (25.2%; 95% CI: 20.7 to 31.5), summer (24.1%; 95% CI: 19.1 to 29.8), and autumn (19.4%; 95% CI: 14.6 to 24.8). The tHcy levels in winter were higher than that compared with other seasons, but tHcy levels were no statistically significant differences in seasonal variation (Table 3). There were no statistically significant differences among mean tHcy levels in winter (12.97 μmol/L, *p* = 0.061), autumn (12.56 μmol/L, *p* = 0.079), and spring (12.97 μmol/L, *p* = 0.131) compared with summer.

**Table 3:** Association between total homocysteine levels and season variation.

Variable	sCAD-homocysteine, μmg/L	95% lower	95% upper	*p*
	Mean (SD)	Confidence limit	Confidence limit	
Season (ref = summer)				
Spring	12.58 ± 2.28	0.986	1.932	0.131
Autumn	12.56 ± 4.11	1.011	2.057	0.079
Winter	12.97 ± 3.07	0.715	1.932	0.061

## Discussion

The findings of the present case-control study provide information on the risk factors for patients with sCAD and the impact of each factor on the risk of sCAD. This study revealed that the level of tHcy in patients with ischemic stroke due to sCAD was mildly higher than that in controls. One of the most important findings is that mild levels of tHcy are strongly associated with sCAD.

The pathogenesis of vascular damage has not been fully elucidated. From the perspective of the vascular anatomy of sCAD, inherited, mechanical stress, or spontaneous CAD of unknown causes all increase the fragility of the arterial wall to trauma. Our findings suggest that mild-hyper tHcy may be one such precondition for vascular wall damage and are consistent with the results previously obtained in patients with sCAD (9, 15). Related studies have shown that elevated tHcy may lead to an increase in the elastolytic activity of elastic tissue dissociation, which may result in premature fragmentation of the arterial elastic fibers and degradation of the extracellular matrix (16–19). Moreover, an additional effect of tHcy on arterial structures is impaired cross-linking of elastin and collagen (20, 21). In summary, combined with the results of this retrospective study, there is sufficient evidence to speculate that in the pathogenesis of sCAD, increased tHcy concentrations may lead to structural abnormalities in the extracellular matrix of the vessel wall, thus increasing susceptibility to triggering factors and ultimately leading to sCAD. The deterioration of arterial connective structures may be one of the events underlying the association between hyperhomocysteinemia and arterial disease. Two experimental reports have shown that the elastin concentration in the arterial wall of hyperhomocysteinemia mini-pigs is decreased, which is a direct or indirect consequence of the tHcy-induced activation of metalloproteases (18)and serine elastases (19).

However, the underlying mechanism of sCAD is elusive. One study (22) suggested that autoimmunity may contribute to the occurrence of CAD through local inflammation and arterial wall disorders. Moreover, tHcy can induce immunoinflammatory pathways (23); tHcy may interact with CAD through two pathways: arterial wall dysfunction and inflammation induction. Moreover, researchers have reported that tHcy can also promote oxidative stress and modulate the levels of other metabolites, which may lead to coronary artery disease or stroke (24, 25). tHcy activates proteolytic enzymes and interferes with the cross-linking of collagen, causing damage to the middle layer of the arterial wall, which is easily torn to form sCAD.

The incidence of sCAD in terms of seasonal variability was significantly different, which is consistent with the results of other studies (26, 27). Similarly, we found that sCAD occurred more often in winter than in spring, summer, and autumn, but the tHcy levels did not significantly differ seasonally. This significant seasonality of the incidence of sCAD suggests that some key pathophysiological factors may be important in the pathogenesis of this disease despite different geographical and climatic influences. Some northern European and American studies reported a relatively high CAD incidence in the cooler seasons, which means that the incidence of CAD peaks in autumn and winter but not in spring (25–26). In the future, studies with larger sample sizes may be able to detect this phenomenon.

Homocysteine has been suggested to be an acute-phase reactant (26, 29). Therefore, increased tHcy levels could be the consequence of cerebral ischemia or an infection, which has been shown to be associated with sCAD (30, 31), rather than a cause of the disease. Additionally, food deprivation increases tHcy levels (31–33). In the acute phase of sCAD, patients continue fasting, and tHcy may therefore increase independently of the ischemic process. However, the role of tHcy in the pathogenesis of CAD remains to be investigated further.

A limitation of this study is the retrospective nature of the analysis, as the tHcy levels were measured after the onset of sCAD, and the Hcy level increases in the acute phase, which may explain the increased tHcy levels. Another limitation is that tHcy in healthy subjects was not independently included in the comprehensive clinical physical examination, so we did not obtain data from healthy subjects. Similarly, metabolic cofactors of tHcy, such as folate and vitamins B6 and B12, were not included in the routine clinical physical examination and were only measured if the therapist or physician deemed them necessary. In particular, however, previous studies have reported that the levels of folate, vitamin B6, and vitamin B12 in patients with sCAD are within the normal range and that there is no difference between patients with ischemic stroke caused by sCAD and those with other causes (10, 14, 15).

In summary, this case-control study confirmed that an increased level of tHcy in ischemic stroke patients was due to sCAD compared with that in controls. Our study suggests that mild tHcy is an important independent risk factor in young Asian adults with sCAD, but further studies are still needed to determine the possible pathomechanism of sCAD. Therefore, we conclude that fasting tHcy levels should be assessed in young Asian patients with sCAD, which plays an important role in the clinical prediction and early intervention of sCAD. Additionally, reducing the levels of tHcy may be important in the clinical intervention of sCAD.

## Data Availability

The raw data supporting the conclusions of this article will be made available by the authors, without undue reservation.
